# The Evaluation of a Low-Cost Colorimeter for Glucose Detection in Salivary Samples

**DOI:** 10.3390/s17112495

**Published:** 2017-11-01

**Authors:** Rocio B. Dominguez, Miguel A. Orozco, Giovanny Chávez, Alfredo Márquez-Lucero

**Affiliations:** 1 CONACyT-CIMAV S.C., 31136 Chihuahua, Mexico; rb.dominguez@gmail.com; 2 Department of Engineering and Chemistry of Materials, CIMAV S.C., 31136 Chihuahua, Mexico; miguel.orozco@cimav.edu.mx (M.A.O.); gio.chavez.itch@gmail.com (G.C.); 3 CIMAV S.C., Ejido Arroyo Seco, 34147 Durango, Mexico

**Keywords:** saliva, low-cost, RGB, *Diabetes Mellitus*, non-invasive

## Abstract

Given the limited access to healthcare resources, low-income settings require the development of affordable technology. Here we present the design and evaluation of a low-cost colorimeter applied to the non-invasive monitoring of *Diabetes Mellitus* through the detection of glucose in salival fluid. Samples were processed by the glucose oxidase-peroxidase enzymatic system and analyzed with the development equipment. A light emission diode of 532.5 nm was used as an excitation source and a RGB module was used as a receptor. A calibration curve to quantify the concentration of salivary glucose (0 to 18 mg/dL) was carried out by relating the RGB components registered with glucose concentrations, achieving a limit of detection of 0.17 mg/dL with a CV of 5% (n = 3). Salivary samples of diabetic and healthy volunteers were processed with the equipment showing an average concentration of 1.5519 ± 0.4511 mg/dL for the first and 4.0479 ± 1.6103 mg/dL for the last, allowing a discrimination between both groups. Results were validated against a UV-Vis-NIR spectrophotometer with a correspondence of R^2^ of 0.98194 between both instruments. Results suggest the potential application of the developed device to the sensitive detection of relevant analytes with a low-cost, user-friendly, low-power and portable instrumentation.

## 1. Introduction

Colorimetric techniques have been extensively applied for chemical and biochemical detection of relevant analytes in food control, environmental, biomedical and educational fields [1,2,3,4,5]. Recently, custom colorimetric systems have been developed, motivated by the simplicity of colorimetric techniques to demonstrate spectroscopic principles, the cost reduction experienced on electronic parts and the development of low-cost manufacturing techniques such as 3D printers. These systems exhibit characteristics such as portability, low power consumption, ease of use and data transmission potential, and they can be assembled with inexpensive materials [1,6,7,8,9,10,11,12]. The excitation of samples in custom colorimetric systems is usually carried out with light emission diodes (LED), which present advantages over conventional light sources such as low power consumption, long lifetime operation, ease of instrumentation and a variety of defined wavelengths in the visible spectrum, which avoid the use of monochromators [13,14,15]. Colorimetric detection has been performed with light sensitive elements such as photodiodes, phototransistors, CCD cameras and recently by RGB detectors given the number of available devices with this codification for color description (e.g., flatbet scanner, smartphone) [16,17,18]. Finally, to control the operation and data acquisition, embedded systems implemented over low-cost programmable platforms such as Arduino, Microchip PIC^®^ and Raspberry^®^ have been implemented to allow autonomous operation avoiding the need of expensive, voluminous and non-portable equipment [6,19,20,21].

One well-established method for medical diagnostics is the colorimetric detection of glucose for *Diabetes Mellitus* (DM) management [22]. DM is a chronic disease characterized by high glucose content in blood caused either by the absence of insulin secretion or by the inability of the body cells to respond properly to the secreted insulin. The Global Report on Diabetes (GRD) has estimated a worldwide increment in DM cases, from 108 million cases in 1980 to 422 million in 2014 [23]. To avoid major complications such as kidney failure, limb amputation, blindness and coronary artery disease, frequent monitoring of blood glucose is essential for DM patients [23,24,25]. Even though there is plenty of cost-effective technology for blood glucose detection, the requirement of painful and invasive sample collection has promoted the development of alternative techniques for non-invasive glucose monitoring such as bioimpedance spectroscopy, Raman and near-infrared spectroscopy [26,27]. Likewise, to replace blood glucose, the analysis of body fluids with glucose content such as tears, urine, sweat and saliva have been proposed as potential non-invasive methods to improve DM monitoring [28,29,30,31]. Within these, glucose content in saliva has attracted attention given the potential correlation between salivary glucose and blood glucose in diabetic individuals as well as the inherent advantages of saliva samples such as non-invasiveness, cost-effectiveness and ease of collection [32,33,34]. However, given the low concentration of salivary glucose, clinical studies to correlate salivary glucose with blood glucose have been carried out with highly sensitive instrumental techniques such as Gas Chromatography [35] and especially UV-Vis-NIR [32,34,36,37] which present disadvantages such as high-cost, non-portability and the need for a trained personal for execution. 

The development of simple and affordable equipment is especially necessary in low and middle-income countries, which experience a lack of basic technologies needed for diseases management incluiding DM. Aditionally, over the past decade the GRD detected a faster increment in DM prevalence for low and middle-income countries which can potentially increase the number of cases and consequently the healthcare burden for these countries [23,38,39]. In this work, the design and development of a custom, low-cost colorimeter is presented. In order to probe the applicability of the device, glucose detection was selected as a standard method and salivary glucose samples were analyzed to prove the sensitivity achieved by the equipment. The proposed low-cost, portable and easy to use colorimeter was able to detect glucose at low concentration in salivary samples with high accuracy, sensitivity and repeatability and can be considered a suitable alternative to reduce the cost of equipment in medical settings with limited resources. 

## 2. Materials and Methods 

### 2.1. Reagents 

Glucose Oxidase type X-S from *Aspergillus Niger* (GO_x_, 128,200 units/g solid), Horseradish peroxidase type II (HRP, 2100 units/mg solid), 4-Aminoantipyrine (4-APP, CAS 83-07-08), phenol (CAS 108-95-2) and D-(+)-glucose (CAS 50-99-7) were purchased from Sigma-Aldrich(Toluca, Mexico). For GO_x_, 50 mM Sodium Acetate Buffer (pH 5.1 at 35 °C) was prepared from Sodium Acetate Trihydrate (CAS 6131-90-4). For HRP, phosphate buffer (100 mM, pH 6.0 at 20 °C) was prepared from monobasic potassium phosphate (CAS 7778-77-0). During the experimental procedures, a stock solution of 0.01 M (180 mg/dL) of D-(+)-glucose was prepared and working solutions within concentrations from 0 to 18 mg/dL were prepared in tridistilled water. For spectrophotometric measurements, standard disposable PMMA cuvettes were obtained from Brand.

### 2.2. Electronic Components

The Arduino UNO, RGB module (ISL29125) and Real Time Clock (RTC) module (TDLH02) were supplied from Sparkfun Electronics (Niwot, CO, USA). The Green Light Emission Diode (LTL2R3TGY3KS-032A) was purchased from Mouser Electronics (Mansfield, TX, USA). The Micro SD (E336755) and 3.5’’TFT touch screen were obtained from Adafruit Industries (New York, NY, USA). The ABS filaments for the 3D impression were supplied by 3D Market (Queretaro, Mexico).

### 2.3. Equipment

For validation, spectrophotometric measurements of all samples were carried out in a Cary 5000 UV-Vis-NIR spectrophotometer (Agilient Technologies, SantaClara, CA, USA). Spectral measurements for the green LED were performed on the CCS100 compact spectrometer (Thorlabs, Newton, NJ, USA). The cuvette for the assays was inserted on a special container designed on SolidWorks^®^ and printed on a CTC 3D printer (Zhuhai CTC Electronics, Zhuhai, China). All coding was performed on a Panasonic Thoughtbook computer with an Intel^®^ Core™ i5-4310U vPro™ processor under Windows 7 operation system. Blood glucose concentration was registered with a commercial Accucheck active glucose self-monitor (Roche, Indianapolis, IN, USA).

### 2.4. Enzymatic Assay for Glucose Detection

**Figure 1:**
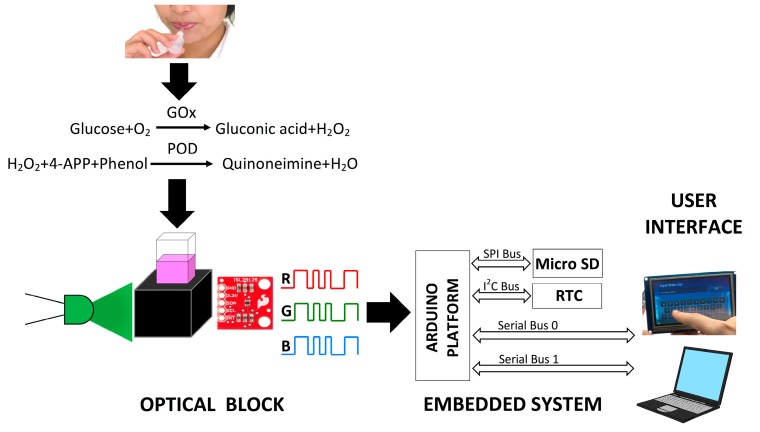
Schematic representation of the implementation of the RGB colorimeter.

Detection of glucose was carried out by the Trinder method, an enzymatic assay based on the GO_x_-HRP system [22]. To perform the assay, an enzymatic solution of GOx (15 U/mL) was prepared in a sodium acetate buffer and a second enzymatic solution of HRP was prepared on a PBS buffer (1 U/mL). The chomophere agent, which promoted color formation, was prepared with 2.6 mM/L of 4-APP and 0.3 mM/L of phenol in 100 mM of TRIS-HCl buffer pH 7.4. In aqueous media, the GO_x_ enzyme in the presence of glucose catalyzes the formation of gluconic acid and hydrogen peroxide (H_2_O_2_). The released H_2_O_2_ acts as an oxidizing agent for the 4-APP+phenol system, which in the presence of HRP forms a purple colored product proportional to the glucose content. The operation of the enzymatic assay is presented in Figure 1. Stock glucose solutions were prepared in tridistilled water within concentrations from 0 to 18 mg/dL and evaluated with the described enzymatic assay. The absorbance produced by the samples was monitored with the Cary 5000 UV-Vis-NIR in the region of 400 to 800 nm, while the equivalent RGB components were extracted by the developed colorimeter. A calibration curve within this range with triplicate measurements was obtained for both methods.

### 2.5. RGB Colorimeter Design

The designed RGB colorimeter is schematically represented by the three blocks shown on Figure 1, namely the optical block, the embedded system and the user interface. The optical block included the assembling of the electronic elements intended for optical detection, such as the green LED of 532.5 nm as excitation source, the ISL29125 RGB module as detector and the required electronic circuit to drive the current for LED operation. The green LED and the RGB module were aligned into a custom 3D holder printed on black ABS filament and designed on Solid Works^®^ for a standard spectrophotometric cuvette of 4.5 mL. The second block of the colorimeter was the embedded system implemented under the Arduino UNO platform, which allows the stand-alone operation of the device. The developed software managed the synchronization and communication of all components intended for detection (RGB module), storage of data (micro SD) and display of results (Touch screen), while the designed PCB allowed the connection of all modules. Data acquisition from the RGB module and operation of the RTC module were controlled through the I^2^C bus of the Arduino UNO platform. The first allowed the capture of digital data to calculate the glucose concentration for each measurement, while the second provided a reference to register the current date and time for each measurement. This information was essential to create a record of glucose measurements, which was stored in the micro SD module through the SPI bus. Finally, two serial buses were activated for interaction with the user. Serial Bus 0 allowed the display of glucose data through a 3.5’’ touch screen while Serial Bus 1 allowed communication with a personal computer. Even PC communication was enabled, the colorimeter was able to operate in stand-alone mode solely with the operation of the optical block, the embedded system and the touch screen as user interface. To perform a measurement, a cuvette with the enzymatic assay described in Section 2.4 was placed on the 3D holder with the green LED pointing out directly over the cuvette while the RGB sensor registered the generated spectral response. This information was converted to a current value expressed in digital data for each component (Red, Green and Blue) and was processed by the embedded system. All samples were analyzed with the developed colorimeter in order to build a calibration curve with the concentration of glucose against the equipment response. The electronic diagrams (schematic and PCB board design) for the colorimeter are presented in Figure S1. In addition, the source files for the electronic system and the 3D holder can be consulted in the supplementary information.

### 2.6. Test with Saliva Samples

To evaluate the ability of the developed equipment to quantify the concentration of glucose in salivary samples, a group of 41 volunteers were recruited to monitor their value of fasting glucose concentration. All subjects gave their informed consent for inclusion before the samples were collected. The participants were asked to arrive in fasting condition with normal oral hygiene. To register blood glucose concentration, a blood sample was taken from each individual and measured with the Accucheck self-monitor glucometer. For salivary glucose, 1 mL of saliva sample collected without any stimulation (e.g., paraffin chewing gum, odor) was stored in 1.5 mL Eppendorf tubes. The unstimulated saliva samples were centrifuged at 4 °C for 10,000 R.P.M. and 200 µL of the resulting supernatant was collected in order to perform the enzymatic assay. After 10 min of incubation, the samples were measured with the commercial spectrophotometer and with the developed colorimeter. 

## 3. Results and Discussion

### 3.1. Development of Colorimeter and Analytical Performance

Body fluids for non-invasive monitoring of DM present a lower concentration of glucose compared with the higher concentration found in blood [16]. Glucose content in blood can be found between 4–8 mM for healthy people and within 2–30 mM for people with a pathophysiological condition [26]. Unlike blood glucose, salivary glucose concentration (SGC) has been studied without a clear cut-off or threshold value for healthy and diabetic individuals. However, several studies support the evidence of higher glucose concentration in diabetic patients as compared with healthy individuals, reporting mean average values of 1.18 ± 0.675 mg/dL [33], 1.23 ± 0.52 mg/dL [40] and 79.4 ± 5.8 µM [34] for SGC in healthy groups. For diabetic groups, mean values of 4.95 ± 2.479 mg/dL [33], 4.22 mg/dL [40] and 187.3 ± 20 µM [34] have been reported. It is also noticeable that even though these studies usually reported mean values, the SGC can be detected at concentrations as low as 0.31 mg/dL and as high as 13.35 mg/dL for uncontrolled diabetics. Thus, these differences were considered for the development of the colorimeter and a range from 0 mg/dL to 18 mg/dL was established as a working range in order to achieve complete detection of SGC.

**Figure 2:**
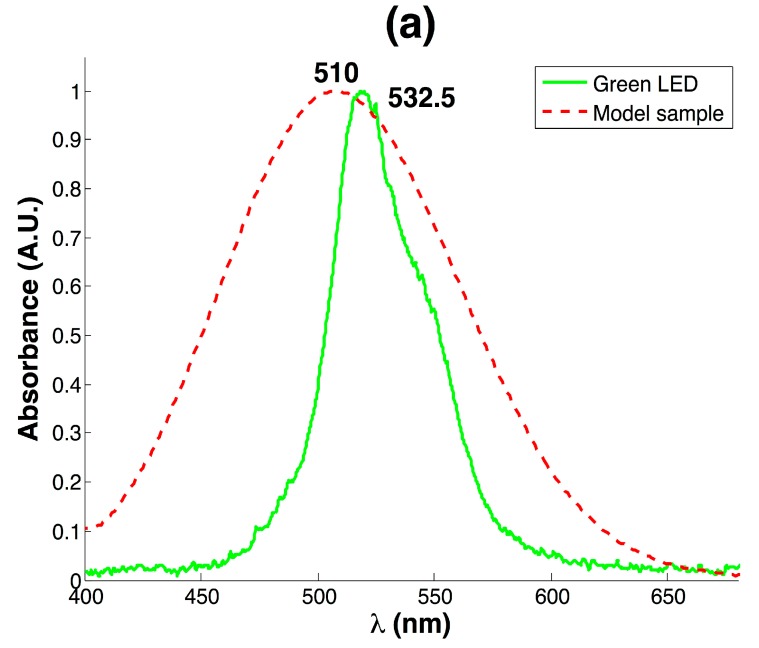

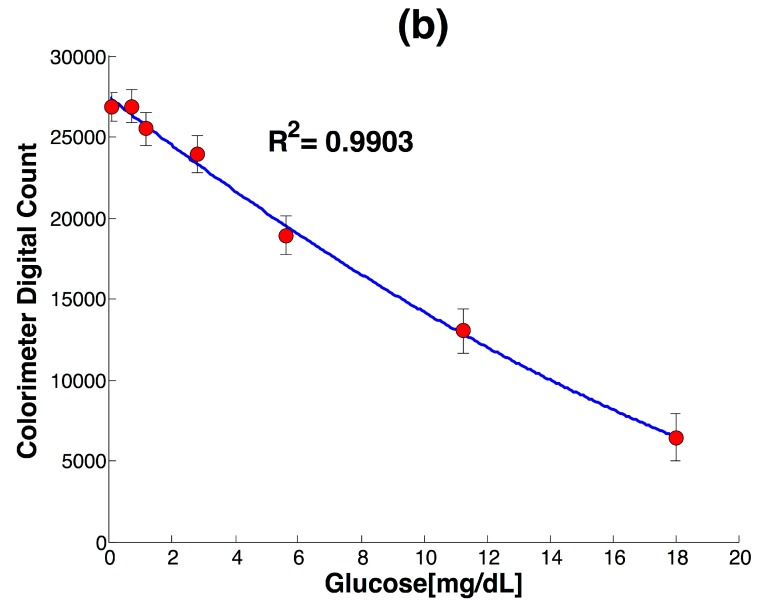
(**a**) The recorded emission specter of the green LED compared with the absorbance specter for 18 mg/dL sample; (**b**) The calibration curve obtained for glucose detection with the RGB colorimeter.

Because the detection of SGC was performed by the GOx-HRP enzymatic system, a purple colored product with higher absorbance towards wavelengths from 505 to 550 nm was expected. To achieve these conditions, a commercial green LED of 532.5 nm was selected as an excitation source and tested with a model sample of 18 mg/dL of glucose. Figure 2a shows the absorbance for the model sample measured with the commercial spectrophotometer compared with the measured emission specter of the green LED. The product of GOx-HRP enzymatic assay exhibited a maximum intensity at 510 nm due to the generated quinone; however, the prevalent wavelength of 532.5 emitted by the green LED fell into a useful high absorbance region, even though it was skewed from the maximum absorbance of 510 nm. However, the affordable cost of the LED compared with conventional light sources as well as its low power consumption (10 mA) support the convenience of the selected device to develop a low-cost instrument. 

Detection was carried out by the ISL29125 RGB module, which allowed the description of received spectral response into current by a three-phototransistor array. The spectral response received by the RGB module was proportional to the quantity of light allowed to pass by the sample, which was related to glucose concentration. The module allowed the conversion of light changes into current values for each channel (Red, Green and Blue), which were later digitalized by a 16 bit ADC with an integration time of 100 m s^−1^. The final response was a digital count result of the integration of the registered current for each sample. In order to compare the absorbance recorded by the commercial spectrophotometer with the digital count equipment response, glucose concentrations within the range of 0 mg/dL to 18 mg/dL were measured in both devices. Glucose concentration was calculated according to the described integrated response for the ISL29125 module, but since the RGB detector was built-in and isolated in the black holder, the main excitation occurred by the green light LED source, which determines a strong response for the Green channel and a low response for the Red and Blue channels. Even though the RGB module allowed the detection of Red, Green and Blue specters, given the strong correlation of the enzymatic assay with the green wavelength, only the green channel was used for detection; however all channels were enabled for further detection if needed. 

From the calibration curve for the developed colorimeter, the analytical figures of merit for the detection of glucose are shown in Table 1. 

The achieved limit of detection (LOD) of 0.17 mg/dL was relevant, since previous studies found low SGC of 0.31 and 0.51 mg/dL in both healthy and diabetic individuals [31,33,40]. Thus, a method with similar or lower LOD was required in order to guarantee glucose detection. Previously, a lack of glucose detection in healthy individuals was presumed as a limitation in the sensitivity of the detection method rather than the absence of glucose. In addition, the achieved LOD was also lower than the detection limit previously reported for the determination of glucose in saliva with the RGB approach [16]. In order to improve analytical performance, several image processing algorithms based on a multivariate approach have been proposed [41,42,43]. Even though these are mainly based on pixel values, the multivariate nature of the measurement based on the description of a glucose concentration by three current values of R, G and B channels can be further explored with chemometric methods such as multivariate calibration, principal component analysis, factorial analysis and even neural network analysis in order to extract meaningful information and improve glucose detection.

**Figure 3:**
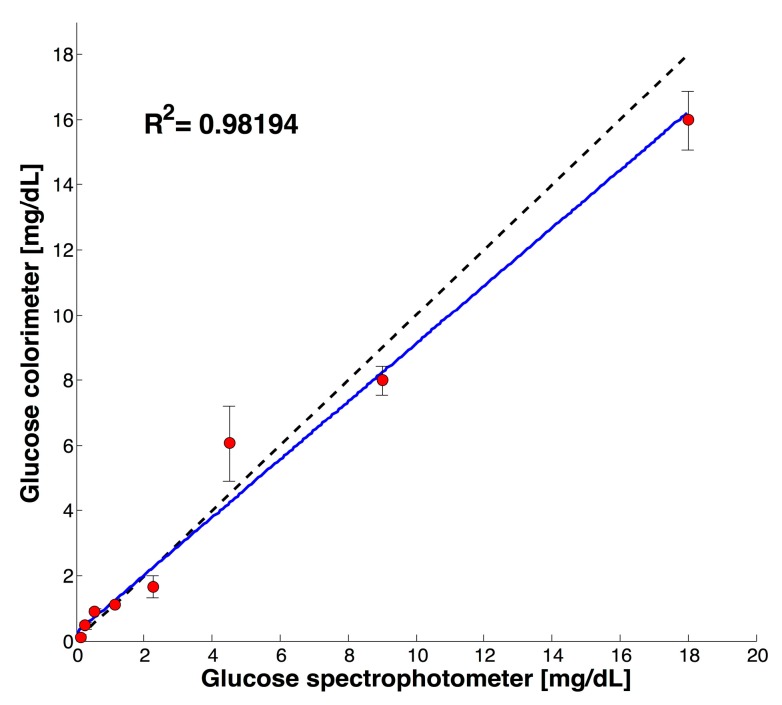
Validation of the developed colorimeter against the commercial spectrophotometer.

Figure 3 shows the validation for results obtained with the commercial equipment Cary 5000 UV-Vis-NIR and the developed colorimeter within glucose concentrations from 0 to 18 mg/dL. A correlation between the two measurements of R^2^ 0.98194 and an average coefficient of variation lower than 5% (n = 3) was achieved for the tested glucose concentrations. The difference of measurements was attributed to the differences between the predominant wavelengths applied to the sample on the commercial spectrophotometer and the developed colorimeter (510 against 532.5 nm). Previously, Anzalone et al. reported a similar behavior during the quantification of chemical oxygen demand with an open source colorimeter designed with a LED with a prevalent wavelength of 620 nm as compared with the required 606 nm [6].

In addition to Figure 3, a set of samples comparing the reference UV-VIS-NIR method against the developed colorimeter is shown in Table 2.

The obtained analytical results for detection and validation with commercial equipment are relevant given the simple design presented and the low-cost of the device. Previous low-cost systems for analytical detection based on RGB description have also been presented as affordable alternatives from optical companies. The main drawback of some of the first devices is the dependency on external systems for management of information, control and detection such as laptops, scanners and smartphones. The inclusion of this equipment involves additional cost, such as power consumption and spare parts, that can be overwhelming for developing regions with limited resources. The commercial devices represent a suitable and attractive alternative for highly sensitive detection of analytes, but based on a locked technology. The development of custom designs adds sensitive detection, flexibility and continuous improvement to previous designs at affordable prices. For example, as presented in Table 3, the total cost for assembling the proposed colorimeter was near $65 USD including the electronic materials and the filament for 3D printing. Moreover, the flexible modular design can support the extension to red and blue LEDs as excitation sources with the same detector at minimal cost. Thus, the low-power consumption, autonomous embedded system implemented over the Arduino UNO platform represents a cost-effective alternative for analyte detection. 

### 3.2. Test with Saliva Specimen

The main objective of this work was applying the developed colorimeter for salivary glucose detection. Salivary samples might be affected by factors such as salivary flow secretion, oral hygiene and oral health. Usually, unstimulated saliva has been preferred for SGC given the diluting and cleaning capabilities of high salivary flow [44]. Poor oral hygiene and oral health issues like *gingivitis* can adversely affect the quality of the sample and the final measurement. Other factors to be considered for salivary samples are the metabolic state of the patient, diet, site of collection, hydration, age, lifestyle and glycemic control [33,37,44]. 

In order to probe the feasibility of SGC with the developed colorimeter, a previous screening test to compare the level of salivary glucose for one healthy volunteer and one diabetic volunteer at fasting conditions and 2 h after glucose intake was performed. Figure S2a shows the increment registered in blood glucose for this preliminary test in both the diabetic and healthy subject, while Figure S2b shows the results for salivary glucose. While the glucose level in the healthy patient showed a moderate increment in both salivary and blood glucose concentration, the diabetic volunteer showed a higher increment in blood glucose which was reflected in salivary glucose content as well. 

**Figure 4:**
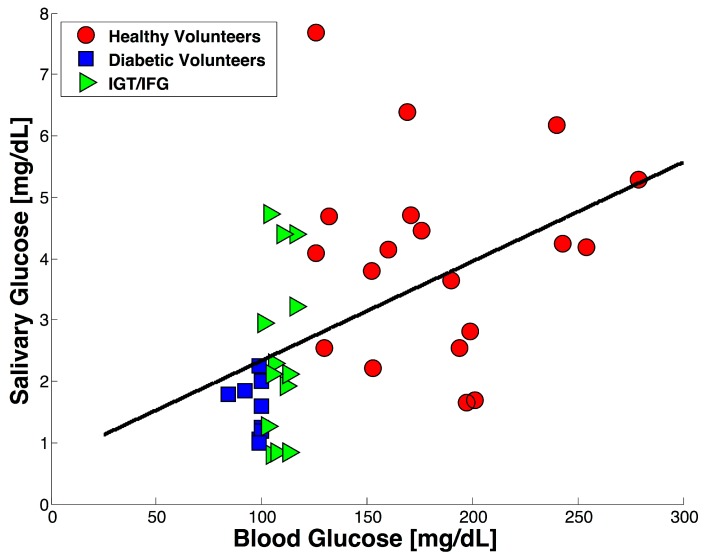
The distribution of salivary glucose concentration against blood glucose concentration for a group of 41 volunteers.

The developed colorimeter was applied to an exploratory study with 41 participants in order to investigate the value of salivary glucose compared with blood glucose in fasting conditions. Blood and salivary samples without stimulation were collected for each volunteer and the obtained results are shown in Figure 4. As expected, the blood glucose values clearly established a difference between the healthy volunteers and the diabetic volunteers. Average blood concentration values of 97 ± 5.5 mg/dL and 183.78 ± 45.17 mg/dL were obtained for the healthy and diabetic group respectively, while the average salivary glucose concentration was found to be 1.5519 ± 0.4511 mg/dL for the healthy group and 4.0479 ± 1.613 mg/dL for the diabetic group. 

Both SGC mean values for healthy and diabetic individuals are in good agreement with the mean values previously reported for salivary glucose detection [33,36,40]. Even though there is no consensus about a cut-off value for salivary glucose concentration, the SGC results obtained for the diabetic group were significantly higher than for the healthy group. The elevated salivary glucose concentration can be attributed to a “leakage” effect on the parotid gland experienced by diabetic subjects when blood glucose levels increase beyond a threshold value [33]. Additionally, according to the current World Health Organization criteria for DM diagnosis, an individual with a blood glucose concentration ≥ 126 mg/dL can be considered diabetic, while individuals with blood glucose higher than 110 mg/dL but lower than 126 mg/dL can be considered affected with impaired tolerance glucose (IGT) or impaired fasting glycaemia (IFG) [23]. Individuals within these glucose values were represented with green triangles and obtained a mean SGC of 2.4509 ± 0.7961, a similar behavior to that observed by Borg et al. [45]. 

Finally, in this work quantification of salivary glucose samples was performed with low-cost equipment. In accordance with the literature, higher glucose concentration was found for diabetic subjects than for non-diabetic subjects but we have extended this knowledge to the field of low-cost instrumentation. Even though glucose was selected as a model analyte, the detection can be extended to other relevant analytes of medical interest besides glucose with similar spectroscopic requirements.

## 4. Conclusions

In this work we present a low-cost RGB colorimeter applied to salivary glucose detection validated against a commercial spectrophotometer. The results showed a good correlation with data obtained by the commercial equipment of 0.98194 R^2^ and a maximum average variation lower than 5% (n = 3). The equipment was applied to the analysis of real samples of saliva in order to discriminate between diabetic and healthy volunteers. The equipment was able to provide accurate information for detection of glucose in real salivary samples, proving the applicability of the instrument to salivary glucose and potentially to other relevant analytes of medical interest with similar spectroscopic requirements. 

## Figures and Tables

**Table 1:** Analytical figures of merit for the developed RGB colorimeter.

LOD	R^2^	STD (n = 3)
0.17 mg/dL	0.9903	5%

**Table 2:** Validation of samples with the reference method.

UV-Vis-NIR [mg/dL]	Colorimeter [mg/dL]	CV (n = 3)
0.56	0.66	4.7%
2.25	2.26	3.8%
9	8.67	4.06%
1.91	1.65	4%

**Table 3:** Average cost of colorimeter development.

Element	Price (USD)
ISL29125 RGB module	7.95
Green LED	0.30
Arduino UNO	10.00
RTC module	8.95
Touch screen	30
Micro SD	7.50
ABS filament	0.4
Total	65.1

## Supplementary Materials

The Supplementary Materials are available online at http://www.mdpi.com/1424-8220/17/11/2495/s1. Figure S1a: schematic design for developed colorimeter, Figure S1b: PCB board for developed colorimeter, Figure S1c: 3D view of designed holder, Figure S2a: Blood glucose record for healthy and diabetic volunteer before and after glucose intake, Figure S2b: Salivary glucose record for healthy and diabetic volunteer before and after glucose intake. The source files for electronic design were provided under the names colorimeter.sch and colorimeter.brd, the sources files for 3D holder can be consulted as holder_cuvette.SLDPRT.
