# Supplementary material for: The Evaluation of a Low-Cost Colorimeter for Glucose Detection in Salivary Samples
Source: Sensors (Basel). 2017 Nov 1;17(11):2495. doi: 10.3390/s17112495 (PMC5713636; doi:10.3390/s17112495)
Supplement: Supplementary file 1 [file sensors-17-02495-s001.zip › Supp_Inf/sensors-226905-supplementary.docx]

Article

Evaluation of Low-cost Colorimeter for Glucose Detection in Salivary Samples

Rocio B. Dominguez^1^, Miguel A. Orozco^2^, Giovanny Chávez^2^ and Alfredo Márquez-Lucero ^3^*

^1^ CONACyT-CIMAV S.C., 31136 Chihuahua, Mexico

rb.dominguez@gmail.com(R.B.D.);

^2^ Department of Engineering and Chemistry of Materials, CIMAV S.C., 31136 Chihuahua, Mexico

miguel.orozco@cimav.edu.mx(M.A.O.); gio.chavez.itch@gmail.com(G.C.)

^3^ CIMAV S.C., Ejido Arroyo Seco, 34147, Durango, Mexico

alfredo.marquez@cimav.edu.mx (A.M.L.)

***** Correspondence: rb.dominguezcruz@gmail.com; alfredo.marquez@cimav.edu.mx; Tel.: +52-614-439-4835

**Supplementary Information**

**S1. Colorimeter design**

**(a)**

**
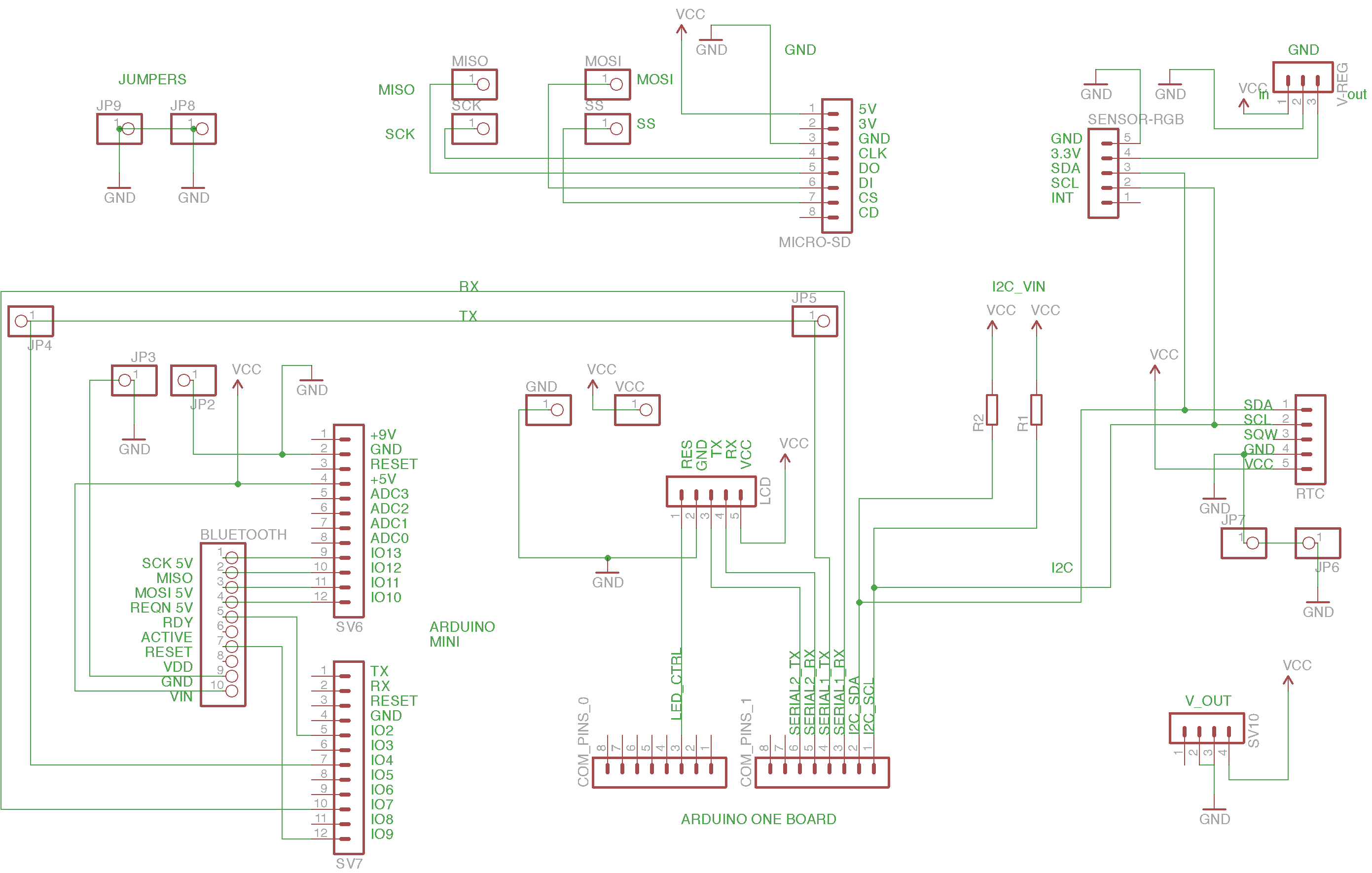
**

**(b)**

**
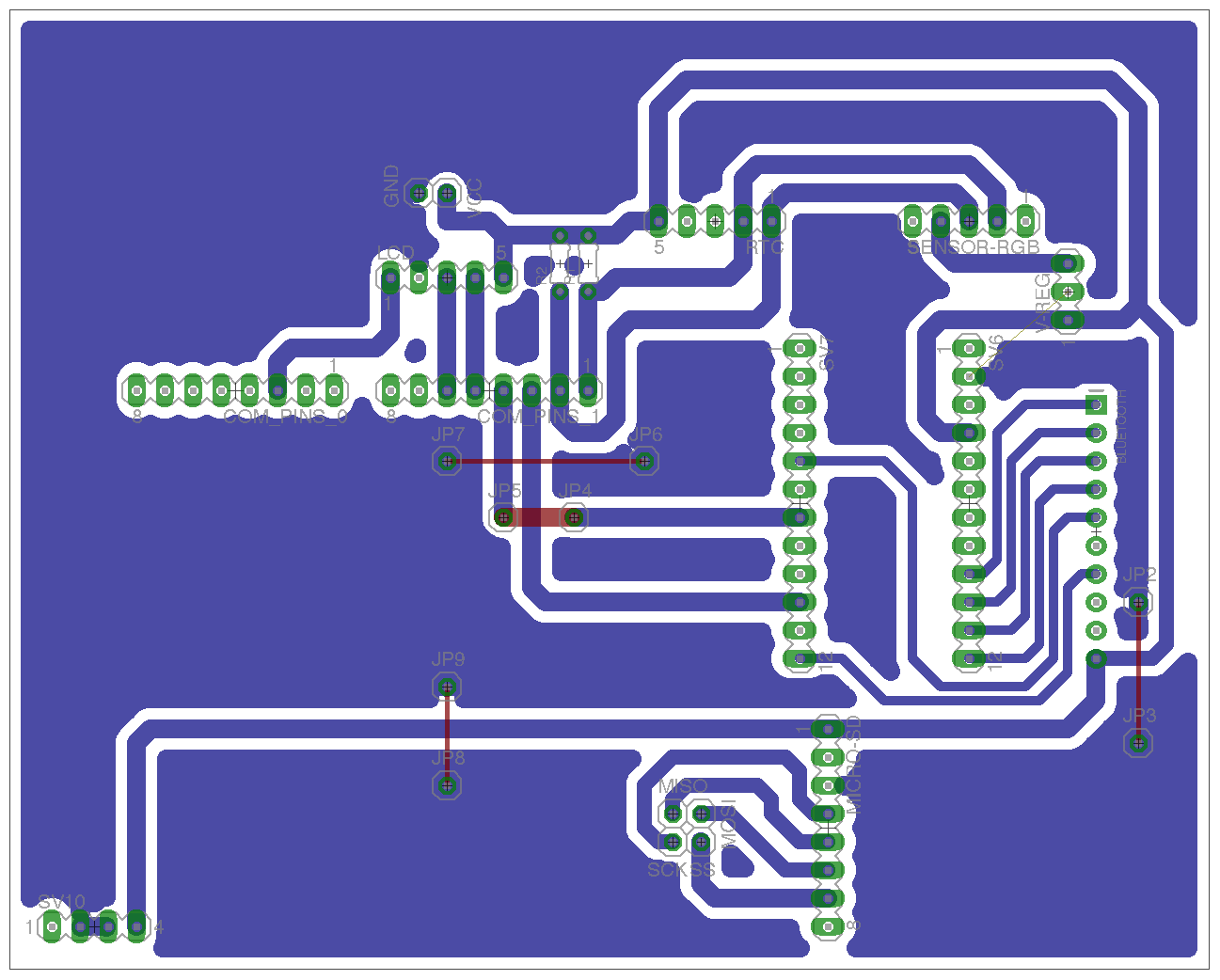
**

**(c)**

**
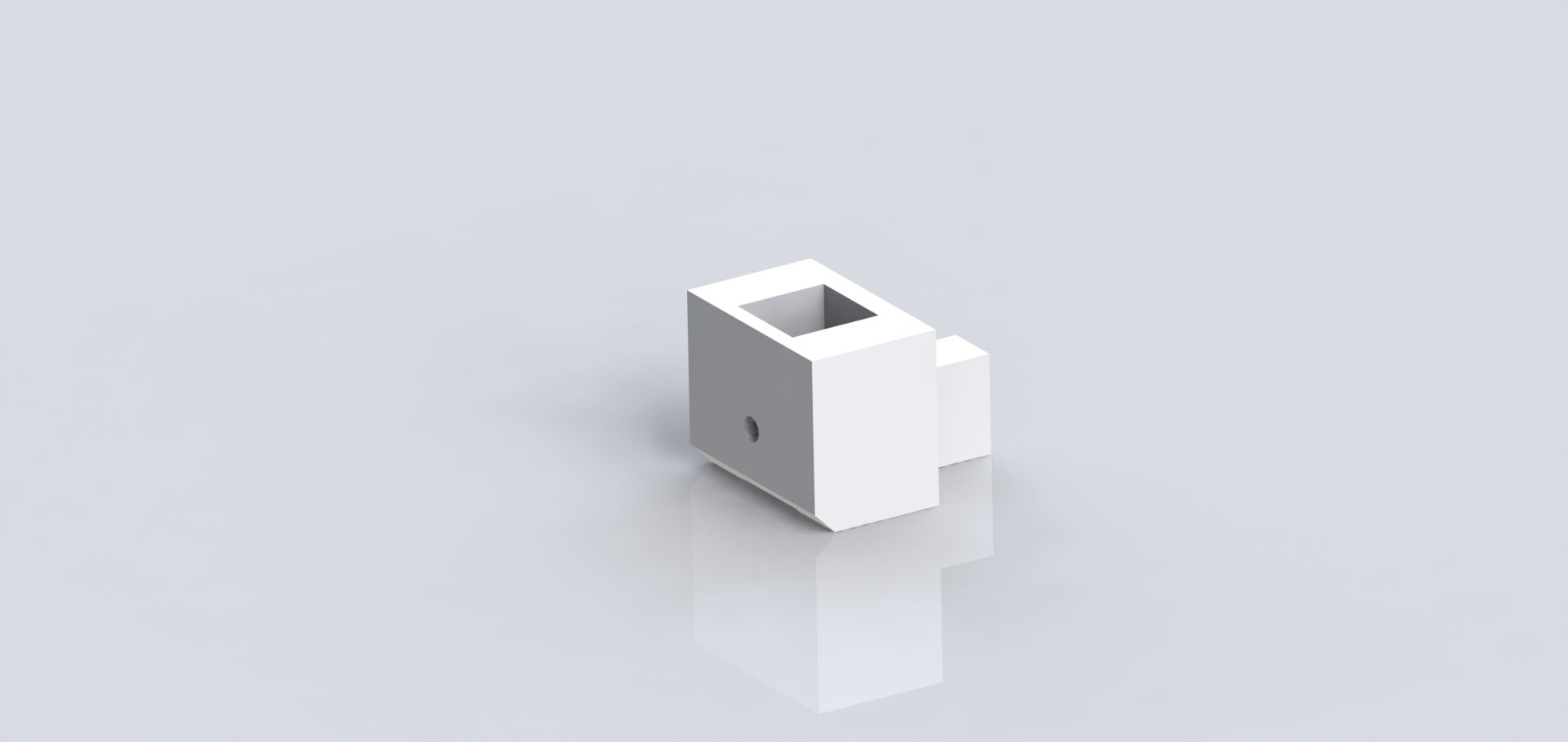
**

**Figure S1.** Figures for electronic design. (a) view for the schematic design of colorimeter (b) view for the PCB design (c) view for the 3D holder

**
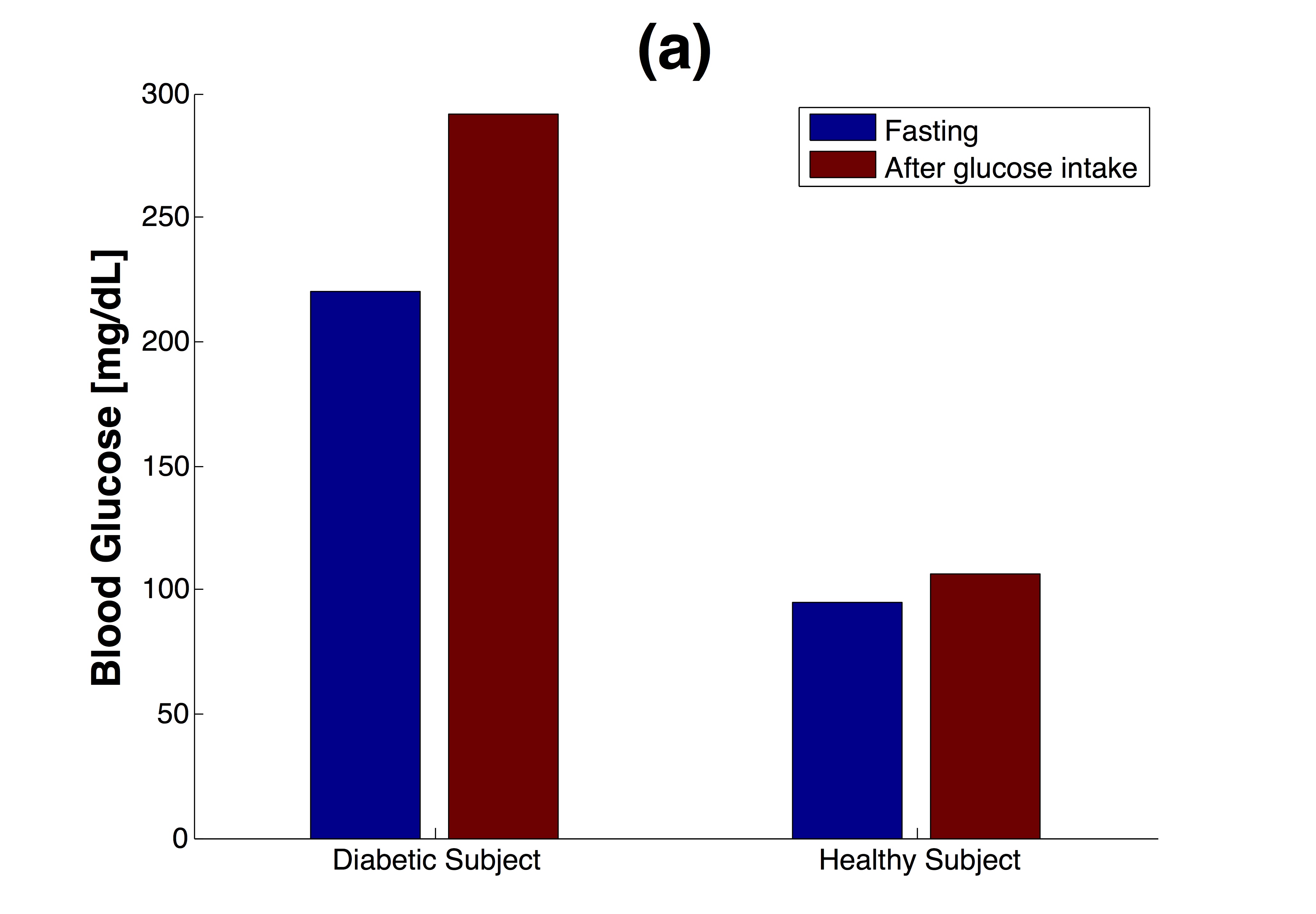
**

**
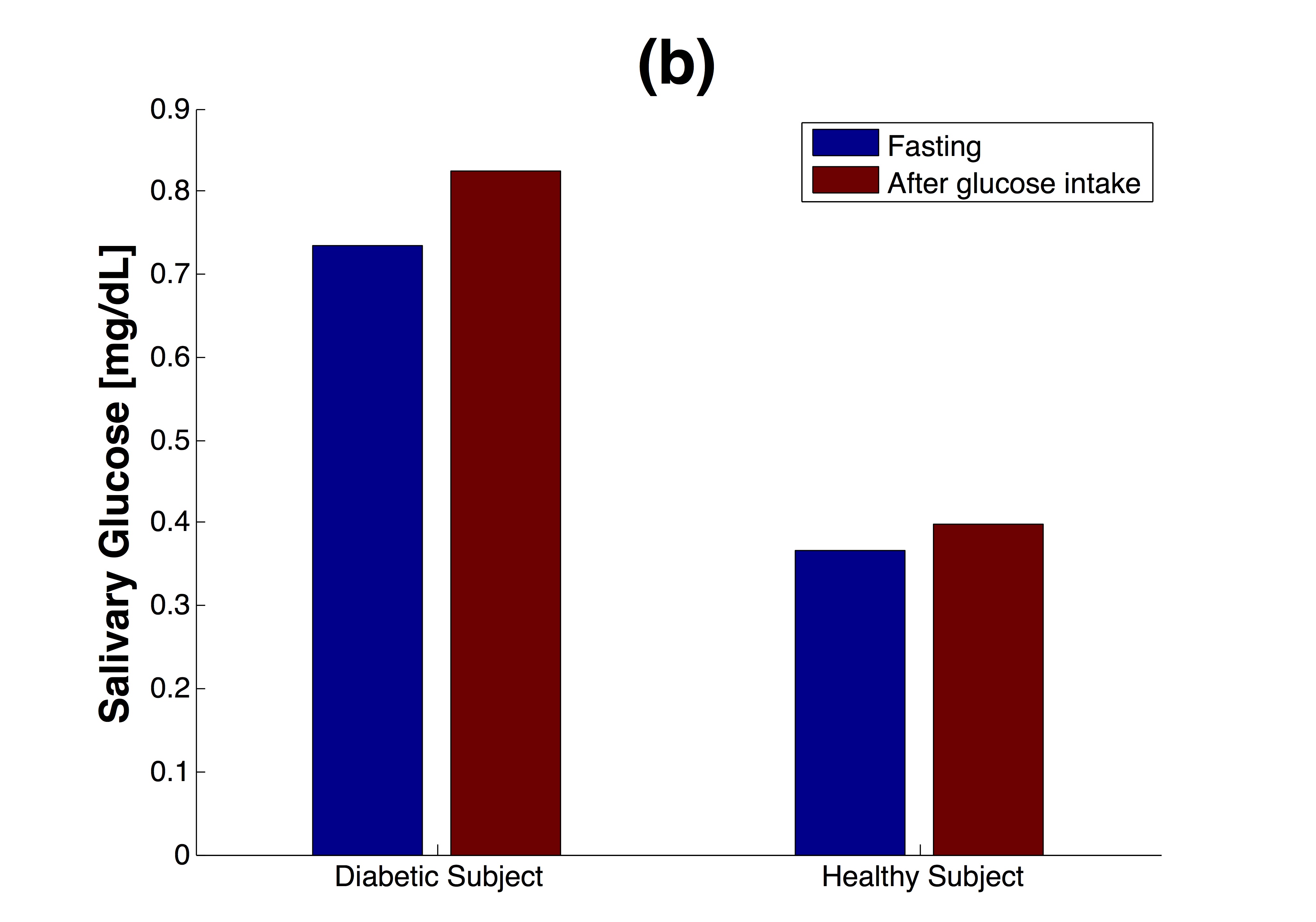
**

**Figure S2. (a)** Tracing of two volunteers at fasting conditions and after glucose intake. Blood glucose recorded for healthy and diabetic volunteer at fasting condition and after 2 hours of glucose intake**. (b)** Tracing of two volunteers at fasting conditions and after glucose intake. Changes in the salivary glucose concentration for the same individuals at fasting condition and after 2 hours of glucose intake
